# DNA-Based Herbal Teas’ Authentication: An ITS2 and *psbA-trnH* Multi-Marker DNA Metabarcoding Approach

**DOI:** 10.3390/plants10102120

**Published:** 2021-10-06

**Authors:** Jessica Frigerio, Giulia Agostinetto, Valerio Mezzasalma, Fabrizio De Mattia, Massimo Labra, Antonia Bruno

**Affiliations:** 1 FEM2-Ambiente, Piazza della Scienza 2, I-20126 Milano, Italy; jessica.frigerio@fem2ambiente.com (J.F.); valerio.mezzasalma@fem2ambiente.com (V.M.); fabrizio.demattia@fem2ambiente.com (F.D.M.); 2 Zooplantlab, Department of Biotechnology and Biosciences, University of Milano-Bicocca, Piazza della Scienza 2, I-20126 Milano, Italy; g.agostinetto@campus.unimib.it (G.A.); massimo.labra@unimib.it (M.L.)

**Keywords:** DNA barcoding, DNA metabarcoding, HTS, herbal teas, food fraud, ITS2, *psbA-trnH*

## Abstract

Medicinal plants have been widely used in traditional medicine due to their therapeutic properties. Although they are mostly used as herbal infusion and tincture, employment as ingredients of food supplements is increasing. However, fraud and adulteration are widespread issues. In our study, we aimed at evaluating DNA metabarcoding as a tool to identify product composition. In order to accomplish this, we analyzed fifteen commercial products with DNA metabarcoding, using two barcode regions: *psbA-trnH* and ITS2. Results showed that on average, 70% (44–100) of the declared ingredients have been identified. The ITS2 marker appears to identify more species (*n* = 60) than *psbA-trnH* (*n* = 35), with an ingredients’ identification rate of 52% versus 45%, respectively. Some species are identified only by one marker rather than the other. Additionally, in order to evaluate the quantitative ability of high-throughput sequencing (HTS) to compare the plant component to the corresponding assigned sequences, in the laboratory, we created six mock mixtures of plants starting both from biomass and gDNA. Our analysis also supports the application of DNA metabarcoding for a relative quantitative analysis. These results move towards the application of HTS analysis for studying the composition of herbal teas for medicinal plants’ traceability and quality control.

## 1. Introduction

Medicinal plants have been used in traditional medicine for centuries due to their therapeutic properties. Although they are mostly consumed as herbal infusions and tinctures, employment as ingredients of herbal and food supplements is increasing worldwide [1]. Consumers’ awareness about healthy diets and their benefits is expanding the botanicals and herbal supplements market [1]. In the United States, the market of herbal supplements is worth over US$7.4 billion per year [2], and the EU market accounts for a value of EUR€ 1.8 billion [3]. Despite this, fraud and adulteration are widespread issues in the herbal and food industry [4]. Medicinal plants are usually sold as herbal tea or as an ingredient, and fraud and adulteration are difficult to identify [5]. The substitution of high-value plants with cheaper ones has been widely reported in the literature [6,7,8]. Moreover, adulteration with toxic plants has also been recorded and may lead to severe health risks [9,10]. These compounds are in high concentration in plants that can be accidentally used in herbal teas [11]. The authenticity of herbal teas can affect the safety of the product and indirectly, the consumers’ trust. Detecting adulteration and identifying the botanical species present in herbal mixtures is fundamental to guarantee the consumers’ safety. Multiple methods, mainly based on morphological and chemical characterization, have been proposed in plant pharmacopoeia. However, these methods fail when the morphological features are lost or when the chemical profiles are shared among congenerics [12]. To identify and confirm the raw ingredients of processed herbal products, quality control involving standard processes can be used to identify specific targeted compounds, but could not detect other non-target plant constituents in herbal samples.

In the last decade, the use of molecular tools for the authentication of food products has drastically increased [13,14]. In this context, the biomolecular analysis of DNA barcoding has become more and more important over time [15]. Although food authentication using DNA barcoding is well-supported and validated when used to identify single species [16,17,18], the characterization of plant complex mixtures and/or processed products is still a challenge. DNA could undergo degradation processes due to industrial treatments. As a consequence, in several processed foods, DNA could be highly degraded and fragmented [19]. To analyze these complex matrices, the DNA barcoding approach was combined with high-throughput DNA-sequencing technologies (HTS), which offers the opportunity to simultaneously sequencing multiple DNA amplicons (DNA metabarcoding) [20]. Nevertheless, DNA metabarcoding still has some limitations, such as amplification biases, accidental laboratory contamination when DNA is in low concentration and the difficulty of ingredients’ quantification [19,21,22]. In our study, we wanted to (i) evaluate if DNA metabarcoding can be a universal and sensitive tool to identify all the species in a product. To accomplish this, we analyzed fifteen commercial products with DNA metabarcoding, focusing on two barcode regions: the nuclear ITS2 and, for the very first time (in a DNA metabarcoding context), to the best of our knowledge, the plastidial intergenic spacer *psbA-trnH*. Additionally, in order to (ii) evaluate the quantitative ability of HTS, we compared the declared occurrence and abundance of plant components in herbal products to the corresponding assigned sequences, creating six mock mixtures of plants in the laboratory starting both from raw plants (biomass) and genomic DNA (gDNA).

## 2. Results

### 2.1. DNA Metabarcoding Characterization of Commercial Herbal Teas

**Figure 1:**
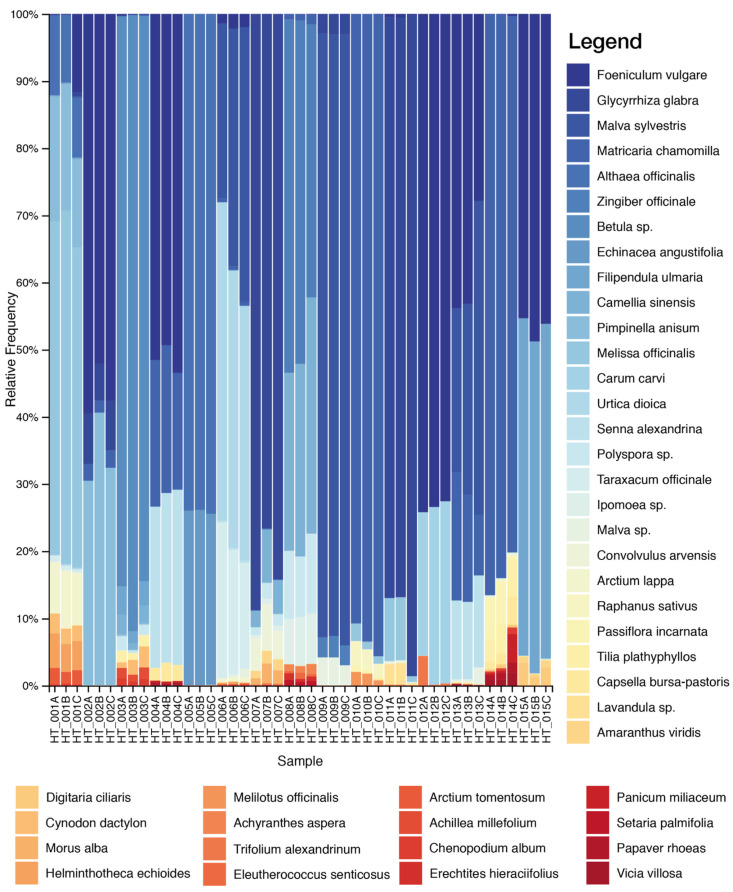
Relative abundance of the plant taxa recovered in the 15 herbal tea products through ITS2 metabarcoding sequencing. Only the taxa present in at least two of the three replicas and in a concentration >1% are shown. The complete list of taxa is reported in Supplementary Table S1.

**Figure 2:**
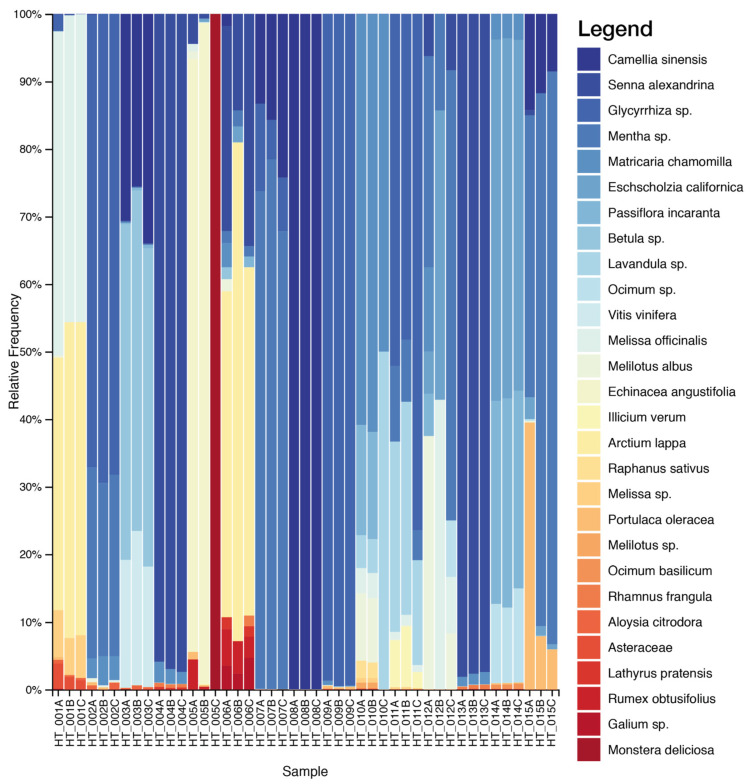
Relative abundance of the plant taxa recovered in the 15 herbal tea products through *psbA-trnH* metabarcoding sequencing. Only the taxa present in at least two of the three replicas and in a concentration > 1% are shown. The complete list of taxa is reported in Supplementary Table S1.

HTS analysis produced about 12,358,533 raw pair reads from the analyzed samples, with an average of 111,338.13 reads per sample (SD = 61,835.85). After quality filtering, merging reads, chimaera removal and clustering, we obtained a total of 508 ITS2 and 235 *psbA-trnH* OTUs. Negative controls for library sequencing were not included in the analysis due to the very low amount of DNA reads. Overall, a total of 83 taxa were identified, of which 35 were found only by the *psbA-trnH* marker and 60 by the ITS2 marker. Considering the species declared on the label, only 12 species out of 53 were identified by both markers (*Arctium lappa*, *Arnica montana*, *Betula* sp., *Camellia sinensis*, *Glycyrrhiza glabra*, *Ilex paraguariensis*, *Matricaria chamomilla*, *Melissa officinalis*, *Passiflora incarnata*, *Paullinia cupana*, *Raphanus sativus* and *Senna alexandrina*) (Figure 1 and Figure 2 and Table 1).

**Figure 3:**
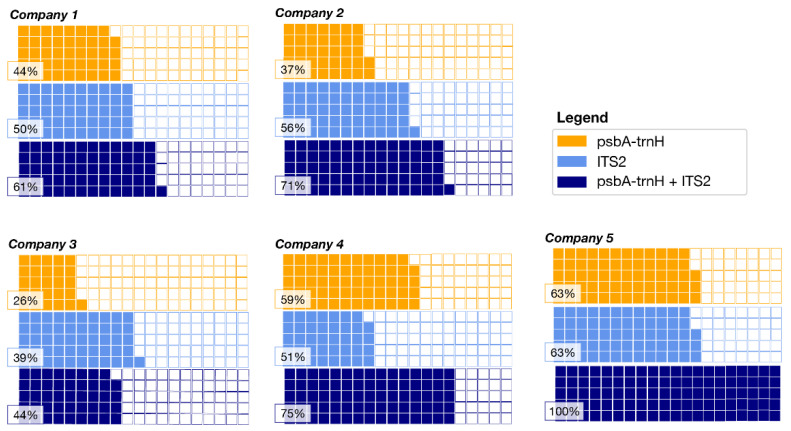
The waffle chart shows, in orange, the percentage of assigned taxa declared in the label for the barcode marker *psbA-trnH*, in light blue, the percentage of assigned taxa declared in the label for the barcode marker ITS2, and in blue, the total percentage using both markers for all the companies. The percentages of assigned taxa are reported in Supplementary Table S1.

Most of the OTUs were assigned to the species taxonomic level, however, in some genera, such as *Mentha*, the low interspecific variability did not allow the species to be identified. On average, 70% (44–100) of the declared ingredients have been identified in the analyzed products (see Table 1). Some products belonging to the same companies (see Table 2) have a higher rate of ingredient identification (e.g., company 5, *n* = 100%) than others (e.g., company 3, *n* = 44%), as can be seen in Figure 3.

Overall, our data reported a mixed composition reflecting, at least in part, the complexity in terms of detected species of the herbal teas. DNA metabarcoding results allowed the detection of the declared species in most of the cases. In all samples, except for HT_013, it allowed the detection of undeclared elements. For example, in the HT_001 sample, we found a high percentage (17%) of *Pimpinella anisum*, a plant typically used in herbal teas but not declared on the label. In the sample HT_002, coming from the same company of sample HT_001 (Company 1), the presence of *Pimpinella anisum* is declared on the label and we found the presence of this ingredient. Additionally, in the HT_012 sample, a high percentage (21%) of a species commonly used for botanicals, *Melilotus officinalis*, was found.

Common infesting herbaceous species, such as *Cynodon dactylon*, *Helminthotheca echioides*, *Chenopodium album*, *Digitaria ciliaris* and *Lathyrus pratensis,* were found in several samples in a percentage range of 2–5%. The presence of such plants could be harmful, as some species could be poisonous [9] or cause allergies [23].

The ITS2 marker appears to identify more species (*n* = 60) than *psbA-trnH* (*n* = 35), with an ingredient identification rate of 52% (39–63) for ITS2 versus 45% (26–63) for *psbA-trnH*. However, the use of both markers has made it possible to almost double the capacity for identifying and assigning species, for some samples (HT_013-HT_015) reaching 100% identification of the declared species on the label. Only 12 species declared on the label have been identified by both markers. It has been noticed that some species are identified only by one marker rather than the other. Specifically, *Eschscholzia californica*, *Mentha* sp., *Vitis vinifera*, *Illicium verum* and *Rhamnus frangula* were identified only with the *psbA-trnH* marker, while the species *Althaea* *officinalis*, *Epilobium angustifolium*, *Foeniculum vulgare*, *Malva* sp., *Pimpinella anisum*, *Solidago virgaurea*, *Taraxacum officinale*, *Urtica dioica* and *Zingiber officinale* were identified only with the ITS2 marker. This result appears to be reproducible considering different samples from different companies. Furthermore, it was also confirmed by the fact that *Althaea* *officinalis* and *Solidago virgaurea* species were present in the mock created in the laboratory and were never recognized by the *psbA-trnH* marker. In order to exclude the failure in ingredient identification due to a lack in the database, for each species, the presence of sequences deposited in the NCBI database (https://www.ncbi.nlm.nih.gov/ accessed on 7 September 2021) was confirmed.

### 2.2. DNA Metabarcoding for Mock Mixtures’ Quantification

**Figure 4:**
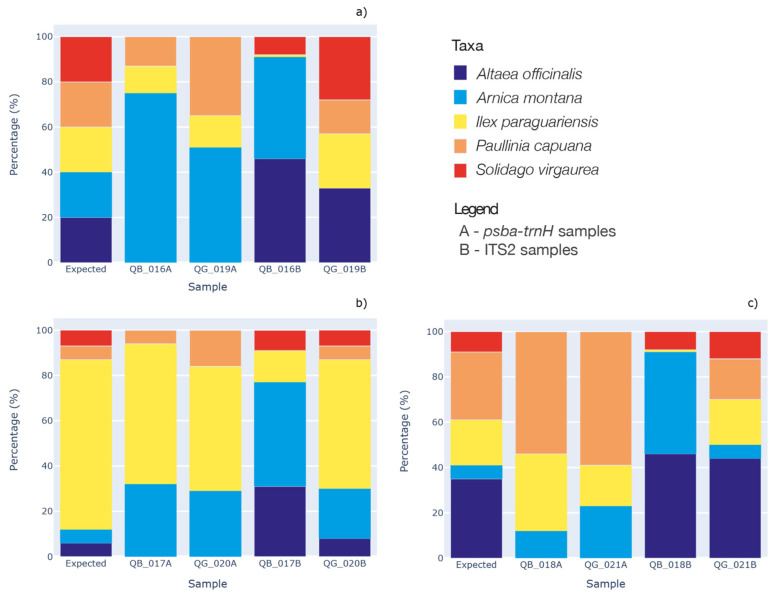
Graphs showing the expected percentages and the percentages obtained after HTS sequencing for the *psbA-trnH* and ITS2 barcode markers both for the mock mixtures created starting from biomasses and genomic DNA. The three different mixture concentrations are shown in (**a**–**c**) panels (see Table 3 for detailed composition). For each panel, Expected: expected composition, QB__A: *psbA-trnH* biomasses, QG__A: *psbA-trnH* gDNA, QB__B: ITS2 biomasses, QG__B: ITS2 gDNA. Percentages are shown in Supplementary Table S3.

As shown in Figure 4, by analyzing both DNA barcode regions (i.e., *psbA-trnH* and ITS2), we were able to identify and correctly assign each plant used for the mock mixtures’ preparation. Nevertheless, through the analysis of the barcode region *psbA-trnH,* we were not able to identify the species *Althaea officinalis* and *Solidago virgaurea*, both in the biomass mixtures and in the genomic DNA mixtures. To confirm this phenomenon, DNA amplification of both species was carried out using both primer pairs (*psbA-trnH* and ITS2). Amplicons were correctly visualized on agarose gel, sequenced and assigned to the species *Althaea officinalis* and *Solidago virgaurea*. Similarly, with the analysis of the ITS2 barcode region, we did not find the species *Paullinia cupana* in the mock mixtures created from the raw plant (biomass). This was probably due to the low yield of genomic DNA (see Supplementary Table S2). Since *Paullinia cupana* was detected in the gDNA mixtures, this result confirms the bias due to the DNA extraction phase.

**Figure 5:**
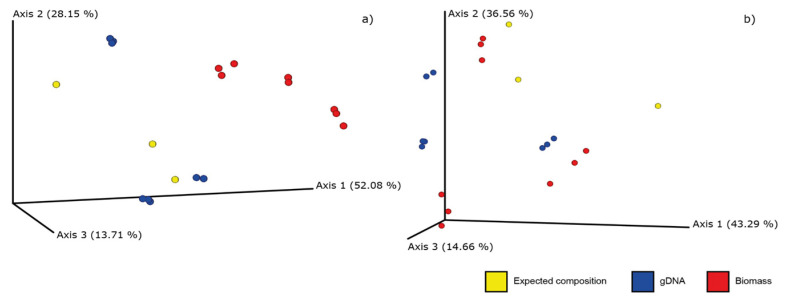
In the PCoA graphs, the yellow dots represent the expected composition samples, the red ones indicate the mock mixtures created starting from the raw plants and the blue ones indicate the mock mixtures obtained from genomic DNA. (**a**) PCoA representation considering ITS2 analysis, while (**b**) *psbA-trnH* analysis.

To determine which mixture strategy (gDNA or biomass) better approximates the expected sample composition, a PCoA analysis with Bray–Curtis distance was performed. As shown in Figure 5, gDNA samples (blue points) appear to be closer to the samples representing the expected composition (in yellow), compared to the mock mixture obtained starting from the raw plants (in red), considering their composition obtained both with *psbA-trnH* and ITS2. To test the difference between the biomass mock mixtures, the gDNA mixtures and the expected composition samples, a PERMANOVA analysis was carried out.

Considering the ITS2 barcode, the comparison between the gDNA group and the expected composition samples group reported no significant differences in composition (q-value = 0.86), while both the comparison between biomass mixture and the expected ones and the biomass mixture and the gDNA group resulted in a significant difference (q-value = 0.012 and 0.003, respectively). Considering the *psbA-trnH* analysis, instead, we observed that gDNA and biomass mixture do not significantly differ (q-value = 0.64), while the comparison of the expected composition group versus biomass and gDNA groups reported a q-value = 0.06 and 0.028, respectively. All the results are available in Supplementary Tables S4 and S5.

## 3. Discussion

Obtaining a representative assessment of complex herbal mixtures is influenced by many factors, including the quality and type of raw material, DNA extraction yield, the DNA barcode(s) choice and a complete reference database [24]. In this study, we took into account these issues, applying a workflow suited to reach the identification of the composition of plant-based processed products.

### 3.1. A Multi-Marker Approach

It is now well-documented that in plants, no single DNA region provides suitable levels of species’ discrimination. The CBOL Plant Working Group (2009) suggest to use two core plastid DNA barcodes, targeting part of the genes rbcL and matK for DNA barcoding [25], often together with a portion of the more variable internal transcribed spacers of nuclear ribosomal DNA (ITS2) [26,27,28], or the plastid intergenic spacer *psbA-trnH* [29].

For this reason, we decided to adopt a multi-marker approach to identify herbal tea composition. Previous studies have used a multi-marker approach for mesozooplankton DNA analysis [30], for dietary analysis of animals [31] and for traditional medicine [32]. As plants have low intraspecific variability, the use of more than one DNA barcoding marker increases the chance of identifying ingredients at the species level. Our analyses confirmed our hypothesis. Considering for instance the sample HT_007, by only using *psbA-trnH,* we were able to assign the genus *Glycyrrhiza* sp., but with the addition of the ITS2 marker, we were able to reach the species level of *Glycyrrhiza glabra*. Additionally, our result showed that *psbA-trnH* and ITS2 markers have a complementary output, with 12 shared taxa identified, but 5 and 9 unique taxa for *psbA-trnH* and ITS2, respectively. A similar result was shown by Arulandhu and colleagues [32], who demonstrated that a multi-marker approach increases the resolution and the quality of the results. It is noteworthy that, even in the mock communities, created ad hoc (known in composition), the differential identification capacity of the two markers was observed. This is not due to the absence of the corresponding sequences in the database: for almost all the species under analysis and for both markers, we checked NCBI database completeness, and for species where the sequence deposited in the database was not present, as in the case of *Paullinia cupana*, we used sequences from our private databases. Thus, these results could suggest that marker choice can significantly affect the outputs. For example, it has already been reported in the literature that primers have a differential affinity for some species, leading to preferential amplification of some taxa, and substantial differences in selectivity among different primers [22,33,34]. Nevertheless, further studies are needed to verify whether this phenomenon is reproducible.

ITS2 marker analysis allowed us to identify a greater number of taxa compared to *psbA-trnH* (60 versus 35). Nevertheless, several species, such as *Melissa officinalis*, *Arctium lappa*, *Matricaria chamomilla*, *Camellia sinensis*, *Senna alexandrina* and *Glycyrrhiza glabra,* were identified by both markers in all samples where they were declared on the label.

This result could be due to the type of sample matrix (such as different parts of a plant or different industrial processing steps) or to the taxa present in the sample analyzed. Ya-Na and colleagues assessed the identification ability of ITS2 and *psbA-trnH* for members of the Apocynaceae family [35]. They reported that ITS2 showed a high identification efficiency of 97% and 100% of the samples at the species and genus levels respectively, and *psbA-trnH* successfully identified 95% and 100% of the samples at the species and genus levels, respectively. It is noteworthy that the barcode combination of ITS2/*psbA-trnH* successfully identified 98% and 100% of samples at the species and genus levels, respectively.

Future studies could expand the number of taxa and analyze different parts of the plant for each taxon, to evaluate whether the matrix can actually affect the amplification preferences of one marker rather than another.

As regards the contaminants detected in the products, since many of them are unique to that product, we can deduce that it is possible that contamination occurred along the supply chain of the manufacturing company. For this reason, HTS analysis can be a method for controlling not only raw materials but also any contamination, which can be both a quality and food safety problem.

### 3.2. Quantitative Ability of High-Throughput DNA-Sequencing

In the scientific literature, there is a debate about the ability of HTS to provide quantitative identification. Lamb and colleagues interpreted the relative OTU abundance as the relative abundance of biomass [36]. The main obstacle concerns all the biases that occur during the analysis, such as DNA extraction, PCR and bioinformatics analysis [34]. In our previous study, we noticed that amplicon DNA metabarcoding efficacy could be biased by the PCR amplification step using “universal” markers, and the occurrence of bias during PCR amplification may cause the inaccurate estimation of quantities. Additionally, Krehenwinkel and colleagues found bias in differential amplification due to priming efficiency during PCR. They suggest using degenerate primers and/or target amplicons with high priming site conservation [37]. In general, the magnitude of difference between the estimated and true values of diversity and abundances vary considering both amplification efficiencies and primer bias, as demonstrated by Kelly and colleagues [38]. Our analysis shows that DNA metabarcoding has a relative quantitative ability, even if there are some biases in the identification of all species. Indeed, in our case, we reported the preferential amplification of some taxa, depending on the primers selected [24,38]. Nevertheless, a relative quantification is achieved, as shown in Figure 3 and demonstrated with PCoA using Bray–Curtis dissimilarity metrics. Moreover, starting from a mixture of gDNA in the ITS2 marker, which is the best one in terms of species identification according to our study, we observed a more accurate quantification compared to the biomass mixture, thus suggesting that the DNA extraction phase could impair the yield of DNA from complex matrices. Nevertheless, using two DNA barcode regions allows overcoming one of the identified biases.

Drawing meaningful conclusions from HTS studies starting from complex matrices is a complex task and strictly depends on understanding the entire process underlying the experimental workflow. In future works, it may be useful to test different parts of the same plant, to verify whether extraction bias can affect the amplification of DNA by one marker rather than the other, both quantitatively and qualitatively. Furthermore, it would be advisable to test different processing levels of a food product, to evaluate the integrity of the DNA and assess whether it can affect the amplification capacity.

## 4. Materials and Methods

### 4.1. Sampling of Herbal Teas and Assembling of Mock Mixtures

Fifteen samples of commercial herbal teas (Table 2) from five different companies were collected from supermarkets.

In order to evaluate the ability of DNA metabarcoding to quantify the ingredients in herbal products, we set up an assay composed both of biomasses and genomic DNA of five plants in different proportions (Table 3). For these mock mixtures, medicinal plant species commonly used as raw material for herbal teas and food supplements were chosen *Althaea officinalis* (roots), *Arnica montana* (flowers), *Ilex paraguariensis* (leaves), *Paullinia cupana* (seeds) and *Solidago virgaurea* (aerial parts). As reported in several studies [39,40], the isolation of DNA can be challenging from some tissues, such as wood and roots. For this reason, different parts of the plant were collected: roots, flowers, leaves, seeds and aerial parts. To test for the difference of quantification of plants in correlation to the sequences obtained before or after the extraction and to identify any bias, these mock mixtures were created with both plant sample quantities expressed as weight of dry material (biomasses) and with different concentrations of genomic DNA (gDNA). In detail, for mock mixtures created by biomasses (QB_016, QB_017 and QB_018), plants were weighed and mixed to obtain the percentages expressed in Table 3. The weights and proportions chosen are consistent with the normal formulation of herbal teas, also in correlation with possible contaminants.

Concerning mock mixtures created by gDNA (QG_019, QG_020 and QG_021), DNA was individually extracted from each dry plant, as indicated in the next section, and individually quantified using a Qubit Fluorometer 4.0 (Thermofisher, Monza, Italy). Each plant species was identified by DNA barcoding analysis. Finally, we prepared different dilutions according to the percentages described in Table 3 of each DNA extract and we composed the artificial mixtures starting from the DNA extracts.

### 4.2. DNA Extraction and Quantification

DNA extractions for herbal teas, positive control and mock mixtures were carried out using the commercial kit DNeasy PowerPlant (QIAGEN, Hilden, Germany), following the manufacturer’s instructions. We started from 50 mg of dry sample material that was homogenized via a mortar and liquid nitrogen; after lysis and wash steps, DNA was eluted in 50 μL of elution buffer and samples were stored at –20 °C. Three technical replicates of DNA extraction were created for each sample, and negative controls of extraction were created. Genomic DNA concentration was evaluated by a Qubit 4.0 Fluorometer (Thermofisher, Monza, Italy). DNA concentrations (ng/μL) for all samples are indicated in Supplementary Table S2.

### 4.3. Libraries’ Preparation and Sequencing

In order to improve the ability to identify all the ingredients at the species level, two universal markers of DNA barcoding were analyzed in this study: *psbA-trnH* and ITS2. ITS2 was selected as it was used in many DNA metabarcoding studies on plants and herbal teas [13,41,42], while *psbA-trnH* was selected because it has a high intraspecific variability [43]. Amplicons were obtained using the same approach described by Bruno et al. [19] with an Illumina adapter (Supplementary Table S6) using puReTaq Ready-To-Go PCR beads (GE Healthcare Life Sciences, Monza, Italy), following the manufacturer’s instructions in a 25 μL reaction containing 1 μL 10 μM of each primer and up to 30 ng of gDNA. PCR cycles consisted of an initial denaturation step for 5 min at 94 °C, followed by 40 cycles of denaturation (30 s at 94 °C), annealing (30 s at 56 °C) and elongation (1 min at 72 °C), and hence, a final elongation at 72 °C for 10 min. Amplicon DNA was checked for concentration by using a Qubit dsDNA HS Assay Kit (Invitrogen, Carlsbad, CA, USA) (Supplementary Table S2) and amplicon length was measured by comparison against QX DNA Size Marker using the Qiaxcel Automatic electrophoresis system (QIAGEN, Hilden, Germany). Samples were sequenced by IGA Technology Services (Udine, Italy). The sequencing was carried out on the MiSeq sequencing platform (Illumina, San Diego, CA, USA) with a paired-end approach (2 × 300 bp).

### 4.4. Bioinformatic Analysis and Data Visualization

Illumina reads were analyzed with QIIME2 (ver. 2020.8; https://qiime2.org/, accessed on 7 September 2021) [44]. After demultiplexing, primers were trimmed, and ITS2 sequences were filtered with a minimum length of 100. Sequences were merged, dereplicated and chimaeras were removed via the de novo method [45]. OTU clustering was applied with a 1.0 similarity threshold. OTUs shared in at least two samples and with at least 250 sequences were kept. The taxonomic assignment of OTUs was carried out via BLAST [46], considering only reference sequences belonging to Viridiplantae rank (TaxID: 33090), adopting an identity of 97% and a coverage of 90%. Taxa bar plots were generated with the QIIME2 dedicated plugin taxa (https://github.com/qiime2/q2-taxa, accessed on 7 September 2021).

To explore the similarity existing between gDNA and biomass mock samples, a PCoA analysis was performed. A Bray–Curtis distance [47] was applied to define the similarity matrix, using the QIIME2 core-metrics plugin (https://github.com/qiime2/q2-diversity, accessed on 7 September 2021).

Statistical differences were calculated by permutation-based ANOVA (PERMANOVA) functions of the beta-group-significance plugin [48], with 999 permutations, considering sample type categories. A PERMANOVA pairwise contrast was also performed.

For each company, waffle charts were generated with the PyWaffle Python package (https://pywaffle.readthedocs.io/, accessed on 7 September 2021) based on the results obtained considering the marker percentage of assigned taxa declared in the labels for *psbA-trnH* and ITS2 barcodes compared with the total percentage with both markers.

## 5. Conclusions

The authenticity of an herbal product is a major concern for consumers, producers, processors and food authorities. In this context, high-throughput DNA-sequencing technologies (HTS) allowed us to detect and identify the plant composition of herbal commercial teas. Although HTS technology has some critical aspects, such as the quality of the extracted DNA [8] or the relative ability to quantify all the ingredients, this study showed the value of the application of HTS analysis for a quality control tool and routine monitoring analysis, from the characterization of the raw ingredients to the final processed products. Furthermore, the use of a multi-marker approach has allowed identifying a greater number of species within a sample, and it is therefore advisable, for future work, to use more than one marker to increase the identification rate. In conclusion, this tool can be applied to a wider range of botanicals to improve the traceability of all products. HTS analysis has such a sensitivity that it can find even small quantities of plants that can be potentially poisonous or harmful to health, so this tool has great potential in quality and safety control in the field of herbal teas. For this reason, it is desirable to implement this analysis by EFSA or other control agencies. As these agencies take long periods to implement analysis, it may be appropriate for companies to start using these tools for preventive control of their supply chain and their products, before it becomes mandatory.

## Figures and Tables

**Table 1:** List of the detected ingredients in market samples, based on DNA metabarcoding assignment. For each sample, the declared species, assigned species for the DNA barcode marker *psbA-trnH* and ITS2 and the company are indicated. The percentage values refer to the relative abundance of HTS sequences for each recognized ingredient. Percentage and plants (indicated as scientific or common name) of declared species are reported as indicated in the commercial label. The chosen taxa in the table were present in at least two out of three replicates.

ID LAB	Declared Species	Assigned Species (*psbA-trnH*)	Assigned Species (ITS2)
HT_001	*Agropyron repens Beauv.* 20%*, Taraxacum officinale Weber* 20%*, Arctium Iappa 15%, Cichorium intybus* 15%*, Melissa officinalis* 15%*, Cynara scolymus* 15%	*Melissa officinalis* 52%*, Arctium lappa* 46%*, Reichardia ligulata* 2%	*Melissa officinalis* 51%*, Pimpinella anisum* 17%*, Althaea officinalis* 10%*, Arctium lappa 8%, Helminthotheca echioides* 4%*, Arctium tomentosum* 2%*, Cynodon dactylon* 2%*, Taraxacum officinale* 3%*, Asteraceae* 2%
HT_002	*Foeniculum vulgare* 20%*, Glycyrrhiza glabra* 20%*, Pimpinella anisum* 20%*, Mentha piperita* 20%*, Citrus sinensis var. dulcis* 15%*, Matricaria chamomilla* 5%	*Glycyrrhiza* sp. 69%*, Mentha* sp. 26%*, Matricaria chamomilla* 4%*, Aloysia citrodora* 1%	*Foeniculum vulgare* 54%*, Pimpinella anisum* 37%*, Glycyrrhiza glabra* 7%*, Matricaria chamomilla* 2%
HT_003	*Camelia sinensis* 20%*, Prunus cerasus* 20%*, Citrus limon* 20%*, Betula pendula* 15%*, Agropyron Repens* 15%*, Vitis vinifera* 10%	*Betula sp.* 49%*, Camellia sinensis* 30%*, Vitis vinifera* 21%	*Betula sp.* 89%*, Camellia sinensis* 2%*, Chenopodium album* 1%*, Cynodon dactylon* 2%*, Filipendula ulmaria* 3%*, Achillea millefolium* 1%*, Polyspora axillaris* 1%*, Tilia platyphyllos* 1%
HT_004	*Senna alexandrina* 40%*, Rhamnus frangula* 20%*, Matricaria chamomilla, Foeniculum vulgare*	*Senna alexandrina* 97%*, Rhamnus frangula* 1%*, Matricaria chamomilla* 2%	*Foeniculum vulgare* 52%*, Matricaria chamomilla* 22%*, Senna alexandrina* 24%*, Capsella bursa-pastoris* 2%
HT_005	*Echinacea angustifolia* 30%*, Citrus x limon, Althaea officinalis, Rosa canina, Hibiscus sabdariffa, Sambucus nigra* 10%	*Echinacea angustifolia 94%, Monstera deliciosa* 4%*, Portulaca oleracea* 1%*, Rumex obtusifolius* 1%	*Althaea officinalis* 74%*, Echinacea angustifolia* 26%
HT_006	*Urtica dioica* 30%*, Arctium lappa* 20%*, Taraxacum officinale, Citrus x limon, Malva officinalis*	*Arctium lappa* 62%*, Senna alexandrina* 24%*, Galium sp.* 4%*, Lathyrus pratensis* 2%*, Mentha sp.* 4%*, Rumex obtusifolius* 4%	*Urtica dioica* 43%*, Malva* sp. 35%*, Taraxacum officinale* 19%*, Foeniculum vulgare* 2%*, Matricaria chamomilla* 1%
HT_007	*Camellia sinensis* 51%*, Mentha* 29%*, Glycyrrhiza glabra* 8.25%, *Mentha piperita* 3.9%, *Aloe vera*	*Mentha sp.* 74%*, Camellia sinensis* 17%*, Glycyrrhiza* sp. 9%	*Glycyrrhiza glabra* 84%*, Amaranthus viridis* 2%*, Camellia sinensis* 5%*, Convolvulus arvensis* 5%*, Ipomoea* sp. 1%*, Morus alba* 2%*, Polyspora axillaris* 1%
HT_008	*Camellia sinensis* 62.9%, *Zingiber officinalis* 22%*,* Peach 1%*, Ginseng* 1%*, Aloe vera*	*Camelia sinensis* 100%	*Camellia sinensis* 30%*, Zingiber officinale* 23%*, Eleutherococcus senticosus* 1%*, Ocimum* sp. 25%*, Polyspora sp.* 10%*, Ipomoea* sp. 7%*, Achyranthes aspera* 1%*, Erechtites hieraciifolius* 1%*, Setaria palmifolia* 1%
HT_009	*Zingiber officinale, Citrus limon, Malva sylvestris, Cymbopogon citratus, Glycyrrhiza glabra*	*Glycyrrhiza* sp. 100%	*Malva sylvestris* 94%*, Glycyrrhiza glabra* 3%*, Ocimum* sp. 2%*, Zingiber officinale* 1%
HT_010	*Matricaria chamomilla* 44.4%*, Melissa officinalis* 22.2%*, Betula pendula/pubescens, Passiflora incarnata, Lavandula officinalis* 5.6%	*Matricaria chamomilla* 51%*, Lavandula* sp. 14%*, Melilotus sp.* 11%*, Melissa officinalis* 6%*, Passiflora incarnata* 16%*, Raphanus sativus* 2%	*Matricaria chamomilla* 93%*, Melilotus officinalis* 1%*, Melissa officinalis* 2%*, Passiflora incarnata* 1%*, Raphanus sativus* 3%
HT_011	*Illicium verum* 27%*, Mentha piperita* 25%*, Melissa officinalis, Glycyrrhiza glabra, Lavandula officinalis, Cinchona officinalis, Gentiana lutea* 2%.	*Glycyrrhiza* sp. 60%*, Lavandula* sp. 25%*, Mentha* sp. 7%*, Illicium verum* 7%*, Melissa officinalis* 1%	*Glycyrrhiza glabra* 90%*, Melissa officinalis* 8%*, Lavandula* sp. 2%
HT_012	*Foeniculum vulgare* 40%*, Illicium verum* 40%*, Carum carvi, Mentha piperita* 9%	*Mentha* sp. 41%*, Eschscholzia californica* 20%*, Melilotus sp.* 21%, *Melissa officinalis* 18%	*Foeniculum vulgare* 72%*, Carum carvi* 27%*, Trifolium alexandrinum* 1%
HT_013	*Senna alexandrina* 40%*, Rhamnus frangula* 15%*, Matricaria chamomilla* 15%*, Foeniculum vulgare* 15%*, Malva officinalis* 15%	*Senna alexandrina* 98%*, Rhamnus frangula* 1%*, Matricaria chamomilla* 1%	*Foeniculum vulgare* 38%*, Malva* sp. 34%*, Matricaria chamomilla* 16%*, Senna alexandrina* 12%
HT_014	*Passiflora incarnata, Eschscholzia californica Cham., Matricaria chamomilla, Tilia platyphyllos, Ocimum basilicum*	*Eschscholzia californica* 53%*, Passiflora incarnata* 30%*, Matricaria chamomilla* 4%*, Ocimum sp.* 13%	*Matricaria chamomilla* 83%*, Passiflora incarnata* 7%*, Panicum miliaceum* 2%*, Papaver rhoeas* 1%*, Tilia sp.* 4%*, Vicia villosa* 1%*, Capsella bursa-pastoris* 2%
HT_015	*Camellia sinensis, Filipendula ulmaria, Foeniculum vulgare, Mentha spicata*	*Mentha sp.* 69%*, Portulaca oleracea* 17%*, Camellia sinensis* 12%*, Eschscholzia californica* 2%	*Filipendula ulmaria* 50%*, Foeniculum vulgare* 47%*, Digitaria ciliaris* 2%

**Table 2:** The ID specimen for all samples, the company of production and the sample typology.

ID LAB	Company	Sample Typology
HT_001	Company 1	Purifying Herbal Tea
HT_002	Company 1	Digestive Herbal Tea
HT_003	Company 1	Slimming Herbal Tea
HT_004	Company 2	Laxative Herbal Tea
HT_005	Company 2	Aromatic Herbal Tea
HT_006	Company 2	Purifying Herbal Tea
HT_007	Company 3	Aromatic Herbal Tea
HT_008	Company 3	Aromatic Herbal Tea
HT_009	Company 3	Depurative Herbal Tea
HT_010	Company 4	Relaxing Herbal Tea
HT_011	Company 4	Digestion Herbal Tea
HT_012	Company 4	Flat Stomach Herbal Tea
HT_013	Company 5	Laxative Herbal Tea
HT_014	Company 5	Sleep Herbal Tea
HT_015	Company 5	Draining Herbal Tea

**Table 3:** The percentages of the plants used for the mock mixture creation. Samples QB_016, QB_017 and QB_018 were created starting from the biomasses of raw plants, and samples QG_019, QG_020 and QG_021 were created starting from the genomic DNA.

Species	Plant Section	QB_016	QB_017	QB_018	QG_019	QG_020	QG_021
*Althaea officinalis*	Roots	20%	6%	35%	20%	6%	35%
*Arnica montana*	Flowers	20%	6%	6%	20%	6%	6%
*Ilex paraguariensis*	Leaves	20%	76%	20%	20%	76%	20%
*Paullinia cupana*	Seeds	20%	6%	30%	20%	6%	30%
*Solidago virgaurea*	Aerial parts	20%	6%	9%	20%	6%	9%

## Data Availability

Data are available at the EBI metagenomics portal (https://www.ebi.ac.uk/metagenomics/), accessed on 7 September 2021, study ID: PRJEB47866.

## Supplementary Materials

The following are available online at https://www.mdpi.com/article/10.3390/plants10102120/s1 Table S1: dataset for bar charts (Figure 1 and Figure 2) and waffle charts (Figure 3) are available in the .xls file, Table S2: gDNA concentrations, Table S3: dataset of HTS sequencing percentages compared to expected samples, Table S4: beta-diversity analysis of ITS2 samples compared to expected samples, Table S5: beta-diversity analysis of psbA-trnH samples compared to expected samples, Table S6: primer sequences.
