# Supplementary material for: DNA-Based Herbal Teas’ Authentication: An ITS2 and psbA-trnH Multi-Marker DNA Metabarcoding Approach
Source: Plants (Basel). 2021 Oct 6;10(10):2120. doi: 10.3390/plants10102120 (PMC8539046; doi:10.3390/plants10102120)
Supplement: Supplementary file 1 [file plants-10-02120-s001.zip › Plants_Frigerio_2021_supplementary_proof.pdf]

plants

DNA-based herbal teas' authentication: an ITS2 and psbA-trnH multi-marker DNA metabarcoding approach

Jessica Frigerio[1], Giulia Agostinetto[2], Valerio Mezzasalma[1], Fabrizio De Mattia[1], Massimo Labra[2], Antonia Bruno[2,*]

[1] FEM2-Ambiente, Piazza della Scienza 2, I-20126 Milano, Italy; jessica.frigerio@fem2ambiente.com (J.F.); valerio.mezzasalma@fem2ambiente.com (V.M.); fabrizio.demattia@fem2ambiente.com (F.D.M.)
[2] Zooplantlab, Department of Biotechnology and Biosciences, University of Milano-Bicocca, Piazza della Scienza 2, I-20126 Milano, Italy; g.agostinetto@campus.unimib.it (G.A.), antonia.bruno@unimib.it (A.B.), massimo.labra@unimib.it (M.L.)
* Correspondence: antonia.bruno@unimib.it

Supplementary Materials: Table S1: dataset for bar charts (Fig. 1 and Fig. 2) and waffle charts (Fig. 3) are available in the .xls file, Table S2: gDNA concentrations, Table S3: dataset of HTS sequencing percentages compared to expected samples, Table S4: beta-diversity analysis of ITS2 samples compared to expected samples, Table S5: beta-diversity analysis of *psbA-trnH* samples compared to expected samples, Table S6: primer sequences

Table S2: gDNA concentrations

ID LAB	ng/µl
HT_001	[5-7]
HT_002	[4-11]
HT_003	[4-5]
HT_004	[2-8]
HT_005	[2-5]
HT_006	[2-4]
HT_007	[10-14]
HT_008	[10-22]
HT_009	[3-6]
HT_010	[3-5]

HT_011	[4-5]
HT_012	[7-27]
HT_013	[2-4]
HT_014	[5-11]
HT_015	[11-15]
QR_016	[6-9]
QR_017	[3-5]
QR_018	[6-8]
QG_019	[31]
QG_020	[23]
QG_021	[36]

Table S2. In the table are indicated the concentration of genomic DNA (ng/μl)

Table S3: Dataset of HTS sequencing percentages compared to expected samples

**Plot**	**Sample**	**Taxa**	**Values**
Plot 1	Expected	*Althaea officinalis*	20
Plot 1	Expected	*Arnica montana*	20
Plot 1	Expected	*Ilex paraguariensis*	20
Plot 1	Expected	*Paullinia cupana*	20
Plot 1	Expected	*Solidago virgaurea*	20
Plot 1	QR_016A	*Althaea officinalis*	0

Plot 1	QR_016A	*Arnica montana*	75
Plot 1	QR_016A	*Ilex paraguariensis*	12
Plot 1	QR_016A	*Paullinia cupana*	13
Plot 1	QR_016A	*Solidago virgaurea*	0
Plot 1	QG_019A	*Althaea officinalis*	0
Plot 1	QG_019A	*Arnica montana*	51
Plot 1	QG_019A	*Ilex paraguariensis*	14
Plot 1	QG_019A	*Paullinia cupana*	35
Plot 1	QG_019A	*Solidago virgaurea*	0
Plot 1	QR_016B	*Althaea officinalis*	46
Plot 1	QR_016B	*Arnica montana*	45
Plot 1	QR_016B	*Ilex paraguariensis*	1
Plot 1	QR_016B	*Paullinia cupana*	0
Plot 1	QR_016B	*Solidago virgaurea*	8
Plot 1	QG_019B	*Althaea officinalis*	33
Plot 1	QG_019B	*Arnica montana*	0
Plot 1	QG_019B	*Ilex paraguariensis*	24
Plot 1	QG_019B	*Paullinia cupana*	15
Plot 1	QG_019B	*Solidago virgaurea*	28
Plot 2	Expected	*Althaea officinalis*	6
Plot 2	Expected	*Arnica montana*	6
Plot 2	Expected	*Ilex paraguariensis*	75

Plot 2	Expected	*Paullinia cupana*	6
Plot 2	Expected	*Solidago virgaurea*	7
Plot 2	QR_017A	*Althaea officinalis*	0
Plot 2	QR_017A	*Arnica montana*	32
Plot 2	QR_017A	*Ilex paraguariensis*	62
Plot 2	QR_017A	*Paullinia cupana*	6
Plot 2	QR_017A	*Solidago virgaurea*	0
Plot 2	QG_020A	*Althaea officinalis*	0
Plot 2	QG_020A	*Arnica montana*	29
Plot 2	QG_020A	*Ilex paraguariensis*	55
Plot 2	QG_020A	*Paullinia cupana*	16
Plot 2	QG_020A	*Solidago virgaurea*	0
Plot 2	QR_017B	*Althaea officinalis*	31
Plot 2	QR_017B	*Arnica montana*	46
Plot 2	QR_017B	*Ilex paraguariensis*	14
Plot 2	QR_017B	*Paullinia cupana*	0
Plot 2	QR_017B	*Solidago virgaurea*	9
Plot 2	QG_020B	*Althaea officinalis*	8
Plot 2	QG_020B	*Arnica montana*	22
Plot 2	QG_020B	*Ilex paraguariensis*	57
Plot 2	QG_020B	*Paullinia cupana*	6
Plot 2	QG_020B	*Solidago virgaurea*	7

Plot 3	Expected	*Althaea officinalis*	35
Plot 3	Expected	*Arnica montana*	6
Plot 3	Expected	*Ilex paraguariensis*	20
Plot 3	Expected	*Paullinia cupana*	30
Plot 3	Expected	*Solidago virgaurea*	9
Plot 3	QR_018A	*Althaea officinalis*	0
Plot 3	QR_018A	*Arnica montana*	12
Plot 3	QR_018A	*Ilex paraguariensis*	34
Plot 3	QR_018A	*Paullinia cupana*	54
Plot 3	QR_018A	*Solidago virgaurea*	0
Plot 3	QG_021A	*Althaea officinalis*	0
Plot 3	QG_021A	*Arnica montana*	23
Plot 3	QG_021A	*Ilex paraguariensis*	18
Plot 3	QG_021A	*Paullinia cupana*	59
Plot 3	QG_021A	*Solidago virgaurea*	0
Plot 3	QR_018B	*Althaea officinalis*	46
Plot 3	QR_018B	*Arnica montana*	45
Plot 3	QR_018B	*Ilex paraguariensis*	1
Plot 3	QR_018B	*Paullinia cupana*	0
Plot 3	QR_018B	*Solidago virgaurea*	8
Plot 3	QG_021B	*Althaea officinalis*	44
Plot 3	QG_021B	*Arnica montana*	6

Plot 3	QG_021B	*Ilex paraguariensis*	20
Plot 3	QG_021B	*Paullinia cupana*	18
Plot 3	QG_021B	*Solidago virgaurea*	12

Table S3. Dataset used to generate graphs in Figure 4 showing the expected percentages and the percentages obtained after HTS sequencing for the *psbA-trnH* and ITS2 barcode markers both for the mock mixtures created starting from biomasses and genomic DNA. The three different mixture concentrations are shown in a), b), c) panels (see Table 3 for detailed composition). For each panel, Expected: expected composition, QB__A: *psbA-trnH* biomasses, QG__A: *psbA-trnH* gDNA, QB__B: ITS2 biomasses, QG__B: ITS2 gDNA.

Table S4: Beta-diversity analysis of ITS2 samples compared to expected samples

Group 1	Group 2	Sample size	Permutations	Pseudo-F	p-value	q-value
Biomass	gDNA	18	999	13.51	0.001	0.003
Biomass	Expected	12	999	9.51	0.008	0.012
gDNA	Expected	12	999	0.008	0.86	0.86

Table S4. Results of the pairwise comparison for beta-diversity analysis considering ITS2 and expected samples.

Table S5: Beta-diversity analysis of *psba-trnH* samples compared to expected samples

Group 1	Group 2	Sample size	Permutations	Pseudo-F	p-value	q-value
Biomass	gDNA	18	999	0.48	0.64	0.64
Biomass	Expected	12	999	2.92	0.04	0.06
gDNA	Expected	12	999	5.67	0.009	0.028

Table S5. Results of the pairwise comparison for beta-diversity analysis considering *psba-trnH* and expected samples.

Table S6: Primer sequences

**Primer name**	**5'-3'**	**Reference**
S2_F	ATG CGA TAC TTG GTG TGA AT	Chen et al., 2010
S2_R	GGA CGC TTC TCC AGA CTA CAA T	
psbA	GTT ATG CAT GAA CGT AAT GCT C	Newmaster and Subramanyam, 2017
trnH	GCG CGC ATG GTG GAT TCA CAA TCC	

Table S6. In the table are indicated the primer sequences for *psbA-trnH* and ITS2 amplification. To every primer was added the Illumina adaptor.
